# Clinical predictors of hospital-acquired bloodstream infections: A healthcare system analysis

**DOI:** 10.51894/001c.123414

**Published:** 2024-09-10

**Authors:** Harjinder Singh, Radhika Sheth, Mehakmeet Bhatia, Abdullah Muhammad, Candi Bachour, David Metcalf, Vivek Kak

**Affiliations:** 1 Internal Medicine Henry Ford Allegiance Health, Jackson, MI, USA; 2 Research and sponsored programs Henry Ford Allegiance Health, Jackson, MI, USA; 3 Infectious Disease Henry Ford Allegiance Health, Jackson, MI, USA

**Keywords:** bacteremia, sepsis, antibiotic stewardship, empiric antibiotics

## Abstract

**INTRODUCTION:**

This study was performed to identify patient factors associated with hospital-acquired bloodstream infections (HABSI) to guide blood culture collection and empiric antibiotic therapy.

**METHODS:**

A retrospective case-control study reviewed the medical records of 350 patients admitted to our health system from September 2017 to April 2020. The patients were 18 years and older and had at least one set of new positive non-contaminant blood cultures collected after 48 hours of admission, defined as HABSI. We developed clinical variables through a literature review associated with it. Univariate relationships between each variable and bacteremia were evaluated by chi-square test. A predictive model was developed through stepwise multivariate logistic regression.

**RESULTS:**

The univariate analysis and stepwise regression analysis showed that temperature >100.4° F (OR: 1.9, CI 1.1 to 3.4), male sex (OR: 1.8, CI 1.0 to 3.0), and platelet count <150,000/µL (OR: 1.8, CI 1.0 to 3.2) were statistically associated with a positive blood culture.

**CONCLUSIONS:**

This model helps identify patients with clinical characteristics associated with the likelihood of HABSI. This model can help guide the appropriate initiation of empiric antibiotics in clinical situations and assist with antibiotic stewardship.

## INTRODUCTION

Hospital-acquired bloodstream infection (HABSI), known as nosocomial bacteremia, is a positive blood-borne infection after at least 48 hours of admission.^1^ HABSI accounts for a significant proportion of sepsis, particularly in critically ill patients, and adds close to a billion dollars annually to healthcare costs.^2,3^ It is also one of the measures under the Centers for Medicare and Medicaid Services (CMS) Hospital-Acquired Condition (HAC) Reduction Program, which impacts hospital reimbursements.^4^ Compared to community-acquired bacteremia, hospital-acquired bacteremia is associated with a much higher mortality.^5^ Common sources of hospital-acquired bacteremia include central or peripheral catheters, catheter-associated urinary tract infections (CAUTI), ventilator-associated pneumonias (VAP), gastrointestinal, skin/soft tissue infections, and post-operative wound infections.^6,7^ Given the large cost and mortality burden, there is a need for prompt identification and management of hospital-acquired bacteremia.

Blood cultures remain the gold standard for diagnosing bloodstream infections, but there remains conflicting evidence on when to draw them.^8^ They are drawn frequently in the hospital, and the decision is based on the ordering clinician’s judgment. The yield of blood cultures in the hospital is often low, and “pan-culturing” leads to unnecessary testing, false positives, and injudicious use of antibiotics.^9^ Overuse of antibiotics leads to complications like antibiotic resistance and *Clostridium difficile* infections.^10–12^ Several risk prediction tools have been developed for use in the emergency rooms (ER), but few are developed to be used at the bedside.^3,13–17^ Our investigation aims to explore the individual risk factors that correlate with the development of hospital-acquired bacteremia. We also developed a model to predict it based on vital signs, laboratory data, and elements of a patient’s history.

## METHODS

### Study Design

The research protocol was approved by the author’s institutional review board. The study members who accessed patient information underwent appropriate training to ensure patient privacy and access to only relevant data per the protocol. We performed a retrospective case-control study, reviewing patients’ electronic medical records admitted to our health system from September 2017 to April 2020.

### Patient Selection

Hospital-acquired bloodstream infection (HABSI) is the presence of one new set of positive blood cultures after 48 hours of admission. We included 540 patients aged 18 years and older with at least one set of positive blood cultures drawn 48 hours after arrival to the emergency department and did not have the same positive culture in the first blood draw. Out of the 540 cases, 481 had new positive cultures. 350 (72.8%) cases were identified as true bacteremia, and 131 (27.2%) were considered contaminants. We defined contaminants as blood cultures where only a single bottle in a set showed growth or those that grew clinically insignificant or non-pathogenic bacteria such as coagulase negative *Staphylococcus*, *Bacillus, Corynebacterium*.^18^

### Clinical Variables

We identified the variables of interest through a literature review before data collection. The data collection was performed by four physicians and entered into a standardized data collection tool. The variables included were: age ≥ 60 years, time to positive blood culture, heart rate ≥ 90/min, systolic blood pressure ≤ 90 mmHg or use of a vasopressor, oral temperature > 38°C (100.4°F), white blood cell count ≥12,000/ µL, lymphocytes ≤ 1000/mm^3^, platelet count < 150,000 /µL, creatinine >2.0 mg/dL, sex, presence of rigors/ chills, mental status, presence of central lines, use of antibiotics (24 hours before collection), need for mechanical ventilation (before drawing blood cultures), the suspected source of infection, and transplant status (if applicable, including bone marrow transplants and solid organ transplantation). This information was obtained by manually reviewing the medical records.

### Statistical Analysis

We first performed a univariate analysis with ANOVA and chi-square tests (where appropriate) to evaluate the relationship between the chosen variables and positive blood culture. These variables were then entered into a stepwise multivariate logistic regression analysis to develop a predictive statistical model. A p-value of <0.15 was chosen as a cutoff to include the final variables in the prediction model. We assigned points to each risk factor weighted approximately by dividing each beta coefficient by 0.32 and rounding up to the nearest integer (such as 1 or 2). Using these points, we attempted to calculate the patient’s score as the sum of these points based on their risk factors. The data were analyzed using IBM SPSS Statistics (Version 27) by the institutional statistician David Metcalf (listed in the authors).

## RESULTS

### Univariate Analysis

A total of 540 cases were reviewed, out of which 481 (89.1%) cases were found to meet inclusion criteria. Of these, 350 (72.8%) were deemed true positives, and 131 (27.2%) were considered contaminants. 260 (54.1%) of patients were male, and the mean age was 64.8 years +/- 15.8 years (SD). We included 78 (16.2%) patients with solid organ or bone marrow transplants, and 13 (16%) of them developed hospital-acquired bacteremia. The characteristics of the cases are shown in Table 1.

**Table 1.** Study Population Characteristics

**Characteristic**	**Total n=481**
Mean Age (+/- SD) years	64.8 ± 15.8
Male, n (%)	260 (54.1)
Female n (%)	221 (45.9)
True Bacteremia n (%)	350 (72.8)
Bone marrow transplant status, n (%)	27 (5.6)
Solid organ transplant status, n (%)	51 (10.6)
Organ transplanted, n (%)	
Heart	5 (1.0)
Kidney	20 (4.2)
Liver	21 (4.4)
Lung	17 (3.5)
Pancreas	4 (0.9)
Small Bowel	2 (0.4)
Organism identified on culture n (%)	
*Candida spp.*	17 (3.5)
*Enterococcus spp.*	64 (13.3)
*Escherichia coli*	54 (11.2)
*Lactobacillus spp.*	6 (1.2)
*Klebsiella spp.*	32 (6.7)
*Pseudomonas spp.*	17 (3.5)
*Staphylococcus aureus*	65 (13.5)
*Streptococcus spp.*	32 (6.7)
Suspected source of infection, n (%)	
*Bone and Joint*	10 (2.1)
*Respiratory*	97 (20.1)
*Intra-abdominal*	160 (33.3)
*Skin/soft tissue*	155 (32.2)
*Other*	43 (8.9)

The most common suspected sources of infection were noted to be intra-abdominal and skin/soft tissue. Univariate correlates of true hospital-acquired bacteremia included male sex, presence of rigors, heart rate ≥ 90/min, lymphocyte count ≤ 1000/mm³, and the suspected source of infection (p<0.05). Thrombocytopenia came very close to statistical significance as a predictor, with a p-value of 0.051. We did not perform a subgroup analysis in the transplant population. (Table 2)

**Table 2.** Univariate Correlates of True Hospital-Acquired Bacteremia

**Variable (assessed within 24 hours before confirming lab culture)**	**True Positive (n=350)**	**False Positive (n=131)**	**p-value**
Sex, Male	203(58)	57(43)	**0.005**
Age ≥ 60 years	234(67)	83(63)	0.517
Time to Lab Culture ≥ 10 Days	106(30)	31(24)	0.174
Lymphocyte count ≤ 1000cells/mm^3^	170(74)	51(60)	**0.018**
Creatinine > 2.0 mg/dL	96(31)	28(25)	0.228
Platelet count < 150,000 per µL	129(43)	34(32)	0.051
White blood cell count ≥ 12,000 cells/µL	121(41)	33(31)	0.082
Fever (temp > 100.4°F)	146(43)	42(34)	0.088
Heart Rate ≥ 90/min	241(69)	74(57)	**0.013**
Systolic blood pressure ≤ 90 mm Hg	36(11)	12(10)	0.864
Lines placed 24 hours before culture collection	132(38)	41(32)	0.283
Antibiotic use 24 hours before culture collection	127(36)	41(32)	0.449
Chills/rigors	31(13)	6(6)	**0.039**
Mechanical Ventilation	73(21)	25(20)	0.799
Mental status change	116(34)	37(29)	0.377
Suspected Source of Infection			**0.009**
*Respiratory*	65(19)	32(27)	
*Bone and Joint*	7(2)	3(3)	
*Intra-abdominal*	129(37)	31(26)	
*Skin/Soft Tissue*	106(31)	49(41)	
*Other (Central nervous system and genitourinary)*	38(11)	5(4%)	

### Clinical Prediction Model

We entered all the variables into the logistic regression analysis to develop a prediction model by assigning points to each variable based on the magnitude of their regression coefficients (see Methods) (Table 3).

**Table 3.** Predictors Identified by Stepwise Logistic Regression Analysis

**Variable**	**Beta**	**Odds Ratio**	**95% CI**	Points*
Intercept	1.85			
Sex, Male	0.28	1.8	(1.0 to 3.0)	1
Time to Lab Culture ≥ 10 Days	0.26	1.7	(0.9 to 3.2)	1
Creatinine > 2.0 mg/dL	0.28	1.7	(0.9 to 3.3)	1
Platelet count < 150,000 per µL	0.30	1.8	(1.0 to 3.2)	1
White blood cell count ≥ 12,000 cells/µL	0.22	1.6	(0.9 to 2.8)	1
Fever (temp > 100.4 F)	0.33	1.9	(1.1 to 3.4)	1
Mental status change	0.23	1.6	(0.9 to 2.8)	1
Suspected Source (Respiratory as Reference Category)				
Bone and Joint	0.01	1.5	(0.2 to 8.6)	0
Intra-Abdominal	0.26	1.9	(0.9 to 3.9)	1
Skin/Soft Tissue	0.70	2.9	(0.8 to 9.9)	2
Other (Central nervous system and genitourinary)	-0.62	0.8	(0.4 to 1.6)	-2

*Calculated by dividing the beta coefficient by 0.32 and rounding to the nearest integer

**Figure 1.**
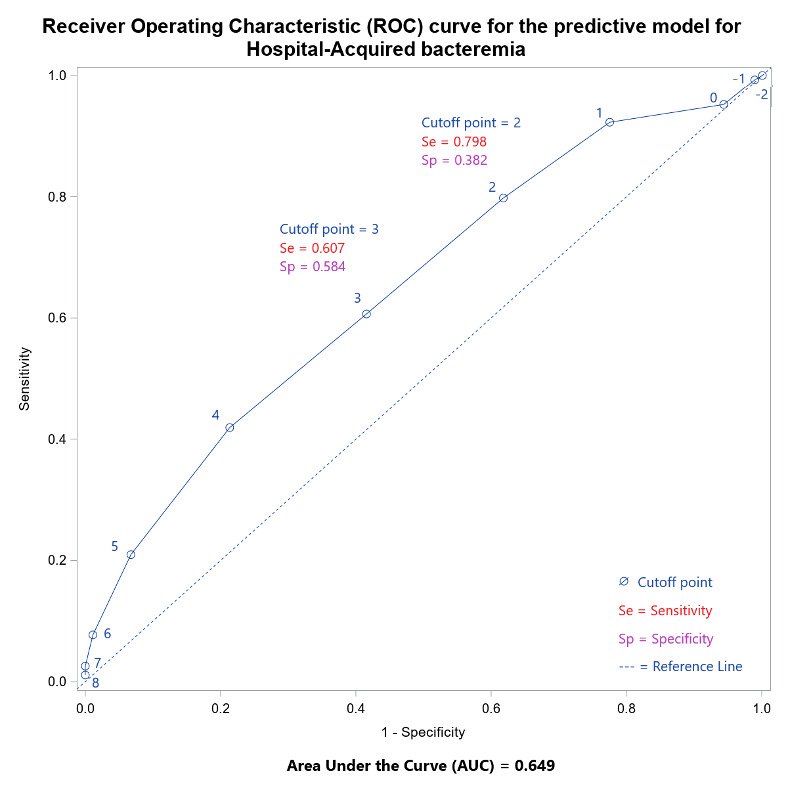
Extrapolation of sensitivity and specificity of the predictive model for hospital-acquired bacteremia.

The model could identify patients at risk of bacteremia by calculating an aggregate score. For example, a patient who did not have any of the risk factors would score zero points. A patient with all the risk factors would get a maximum score of 9 and be considered at a higher risk of bacteremia. Choosing a cutoff score of 2 led to identifying true positives with a sensitivity of 79.8% but a specificity of 38.2%. Intending to identify as many true positive blood cultures as possible, we chose the cut-off point 3, which produced a sensitivity of 60.7% and a specificity of 58.4% (Figure 1).

## DISCUSSION

### Predictive variables

Our study is a retrospective analysis of hospitalized patients who developed HABSI. We looked at variables identified by the literature review and collected this data from the patient records. Through univariate analysis, we identified five variables predictive of true bacteremia, which included male sex, presence of rigors, heart rate ≥ 90/min, lymphocyte count ≤ 1000/mm^3^, and suspected source of infection (p<0.05). We attempted to develop a prediction model for hospital-acquired bacteremia using all the variables in a stepwise logistic regression. Our model seemed to have a fair predictive value, as seen by an area under the curve (AUC) of 0.65.

### Comparison with existing literature

Our sample size was 350, smaller than other retrospective analyses for predicting HABSI.^16–19^ Prediction tools for bacteremia are a concept that has been introduced previously. Several ER-based risk calculators exist for predicting bacteremia,^13,15,20^ but relatively little data is available regarding risk factors for developing HABSI.^3,21^ Bates et al. performed a prospective cohort study of 1516 bacteremia episodes in patients to develop and validate a predictive model identifying HABSI.^3^ Their study identified the following as independent predictors of bacteremia through univariate analysis: the presence of intravenous drug use, shaking chills, major comorbidity, temperature ≥ 38.3°C, hypotension, an acute abdominal examination, leukopenia or leukocytosis, and band count >10%. While we did not consider the patients’ preexisting comorbidities in our univariate analysis, our results found that chills were significantly predictive of bacteremia.

Chills have been associated with infections, especially bacteremia.^22^ Most risk calculators have included rigors as a variable.^3,13,15,16,20^ Shapiro et al. derived an ER decision-making tool using 13 predictors, including chills, and their model had a sensitivity of over 95%.^13^ Lee et al. also looked at risk factors for community-acquired bacteremia. Their multivariate analysis found rigors to be the most significant predictor, with an odds ratio of 13.7 (95% CI, 4.47-42.0).^20^ In the present study, we also found shaking chills/rigors to be predictive, supporting the previous studies’ findings.

In previous studies, leukocytosis was noted to be a predictor of bacteremia.^23.24^ Mellors et al.^3^ defined leukocytosis as a white cell count greater than 15,000/mm^3^, and Weinstein et al. found that a WBC <4000/mm^3^ or ≥ 20,000/mm^3^ was significantly associated with bacteremia. Both studies also found the presence of intravenous catheters to be associated with true bacteremia. The association between lymphopenia with bacteremia and critical illness in the emergency department and intensive care unit has been well described.^23–25^ Our study only found lymphopenia (lymphocyte count <1000/mm^3^) to be an independent predictor.

Studies have shown that bacteremia from an unknown source of infection is associated with higher mortality. A study from Denmark looked at the association of the source of infection with the 30-day case fatality rate (CFR) in patients with community-acquired bacteremia.^26^ The study found that higher CFR was seen in patients with an unknown source of infection. In their study of bacteremia of unknown origin, Leibovici et al. found that mortality in unknown sources was 44% versus 22% in those with a recognizable source.^27^ Our study was not designed to look at the mortality, but the suspected source of infection was a significant predictor of bacteremia. Patients with bone and joint infections and skin/soft tissue infections were more likely to have positive blood cultures. On the contrary, patients with central nervous system and urinary tract infections were less likely to be truly bacteremic.

Male sex was associated with a higher risk of HABSI in our analysis. Male sex is well described possible risk factor for sepsis, with annual relative risk of 1.3 times of that of women.^28^ The sex differences seem to be related estrogen and testosterone influencing immune function leading to a greater preponderance of sepsis in males.^29^

### Who Should Get a Blood Culture?

Based on our model, using a cut-off value of 3 points has the most reasonable sensitivity (61%) and specificity (58%) for identifying true bacteremia. A lower cut-off of 2 points yields a higher sensitivity of 79% at the cost of much lower specificity. The risk of bacteremia in a patient with a score of 2 or more is high, and this population may benefit from having a high clinical suspicion and a low threshold for drawing blood cultures. Patients who develop fever and chills 48 hours after hospitalization may benefit from close observation so that the blood cultures are drawn and possible sources of infection are investigated and treated. We also argue that close monitoring should be directed to patients with skin and intra-abdominal infections as these pose a higher risk of true bacteremia. These sensitivity and specificity values are relatively low as seen in our analysis. The specialty of infectious disease majorly involves using the overall clinical picture on deciding an appropriate antimicrobial treatment. Unlike many other non-infectious pathologies, the confirmatory test in infectious diseases involves of isolation of a particular organism or its components, which is not very time-sensitive. This article reflects on that conundrum and tries to associate particular variables with the possibility of the bacteremia.

### Clinical implications

With the advancing integration of machine learning with healthcare, newer AI-based prediction models may be designed to aid physician decision-making. Mahmoud et al. reviewed 7157 patients with positive blood cultures^16^ to develop a machine learning-based prediction tool. While the clinical judgment of the physician cannot be completely replaced with these tools, they can at least be designed to aid clinical decision-making at the bedside.

Hospital-acquired bacteremia is an important driver of healthcare costs.^30^ Blood cultures are frequently drawn inappropriately in the hospital setting. This often leads to overuse of antibiotics and subsequent complications, including *C. difficile* infection and antibiotic resistance. The SARS-CoV-2 pandemic has led to increased emphasis on antibiotic stewardship and judicious use of resources. The cost of performing a blood culture at our health system is $194, and each case of CLABSI costs the system ~ $45,000. This study aimed to design a predictive model for diagnostic stewardship, reduce avoidable antibiotics, and improve resource allocation.

### Limitations

Our study has a few important limitations. This was a retrospective study, and although we followed a standardized protocol, reviewer data was not confirmed by an independent source. We also reviewed a small sample size of patients compared to other studies. We recognize that the model with a cutoff of 2 leads to good sensitivity but poor specificity. A cutoff of 3 improves on this but with a trade-off of imperfect sensitivity. Lastly, but most importantly, our model was not prospectively validated on other data, which limits generalizability and requires further prospective studies.

## CONCLUSIONS

This analysis suggests that fever and chills/rigors are strong predictive markers for HABSI. Other clinical factors may aid the clinical suspicion but do not correlate strongly with the possibility of bacteremia. They may be used in an appropriate clinical situation with a combination of markers warranting further management. This may aid in antibiotic stewardship and help clinicians obtain blood cultures and administer empiric antibiotics appropriately.

### Conflict of Interest

None
